# Electrochemical Detection of Anti-Breast-Cancer Agents in Human Serum by Cytochrome P450-Coated Carbon Nanotubes

**DOI:** 10.3390/s120506520

**Published:** 2012-05-18

**Authors:** Camilla Baj-Rossi, Giovanni De Micheli, Sandro Carrara

**Affiliations:** EPFL—École Polytechnique Fédérale de Lausanne, MXG 321, Station 12, Lausanne CH-1015, Switzerland

**Keywords:** cytochrome P450, carbon-nanotubes, electrochemical drugs detection, breast-cancer, human serum, personalized therapy

## Abstract

We report on the electrochemical detection of anti-cancer drugs in human serum with sensitivity values in the range of 8–925 nA/μM. Multi-walled carbon nanotubes were functionalized with three different cytochrome P450 isoforms (CYP1A2, CYP2B6, and CYP3A4). A model used to effectively describe the cytochrome P450 deposition onto carbon nanotubes was confirmed by Monte Carlo simulations. Voltammetric measurements were performed in phosphate buffer saline (PBS) as well as in human serum, giving well-defined current responses upon addition of increasing concentrations of anti-cancer drugs. The results assert the capability to measure concentration of drugs in the pharmacological ranges in human serum. Another important result is the possibility to detect pairs of drugs present in the same sample, which is highly required in case of therapies with high side-effects risk and in anti-cancer pharmacological treatments based on mixtures of different drugs. Our technology holds potentials for inexpensive multi-panel drug-monitoring in personalized therapy.

## 1. Introduction

The rising demand for the development of personalized therapy has recently stimulated significant research in investigating electrochemical biosensors based on cytochrome P450 for detection of drugs and other chemical compounds [1]. The detection mechanism of these enzymatic amperometric biosensors is the measurement of the current produced at the electrode surface due to the redox reaction of the enzyme when a substrate is present in the sample. Cytochrome P450 enzymes (CYPs) have widely been used as recognition elements for the construction of amperometric biosensors [1,2] due to the ability of these enzymes to metabolize a wide range of endogenous substances and exogenous compounds, such as drugs and environmental toxins [3]. A cytochrome P450 biosensor is a promising technology that can provide quick measurements for concentrations of drugs and metabolites with good selectivity, accuracy, sensitivity and low-cost equipment. The immobilization of CYP onto the electrode surface has to be accurately controlled in order to obtain a high probability for the protein to be attached to the electrode in a proper orientation so that the electron transfer from the active site of the enzyme is optimized [2,4].

Several attempts have been made to measure drug concentration with P450-based systems [1,2,5–8], but the development of a biosensor capable of measuring a drug mixture is still an open problem. Multiple drug detection with cytochromes P450 is a challenging task because CYP isoforms are not selective for a single compound, and are subject to several atypical kinetic features [9,10] that can affect the protein efficiency according to the concentration and the drug molecules present in the sample.

Different kinds of nano-structures, such as nano-particles, have been extensively employed for improving the CYP-biosensors selectivity and sensitivity [11–16]. Carbon nanotubes (CNTs) have been recognized as a very promising layer for enhancing electron transfer [17], thanks to their electrical and electrochemical properties, which make them suitable to be integrated into biological sensors. For these applications, carbon nanotubes exhibit some advantages: small size with large surface, high conductivity, high chemical stability and sensitivity [18], high electrocatalytic effect and a fast electron-transfer rate [19]. Recent studies demonstrate that CNTs can enhance the electrochemical reactivity of proteins or enzymes with retention of their biocatalytic activity [17,20].

In this work we develop biosensors based on microsomal cytochrome P450 and nanostructured with multi-walled carbon-nanotubes to electrochemically detect drugs used in the treatment of breast-cancer. In order to investigate the feasibility of P450-based sensors for clinical applications, we focused on chemotherapy treatments for breast cancer. In many cases, chemotherapy medicines are given in combination, *i.e.*, two or three medicines administered at the same time [21]. These combinations are known as chemotherapy regimens [22]. After having considered the pharmacological range for various drugs, we selected four compounds: cyclophosphamide, etoposide, ftorafur, and ifosfamide. These four drugs have been chosen because they have sufficiently high and wide pharmacological ranges, which are compatible with the detection limit of the measurement setup.

For the specific detection of these drugs, we employed three different cytochrome P450 isoforms, the CYP1A2, CYP2B6 and CYP3A4; recent studies [23,24] suggest that cytochrome P4503A4 and CYP2B6 may be the major enzymes catalyzing the 4-hydroxylation of cyclophosphamide and ifosfamide; CYP1A2 is known to catalyze the biocativation of ftorafur [25,26]; other studies reported that CYP3A4 and CYP1A2 enzymes are involved in etoposide *O*-demethylation [27].

The main contributions presented in this paper include: (1) a model for describing the protein absorption onto CNTs, confirmed by numerical simulations as well as scanning electron microscopy analysis; (2) drugs detection within the therapeutic range in human serum; and (3) the detection of two drugs present in the sample at the same time. The results demonstrate that the nanostructure of the deposited enzyme and CNT on the electrodes enables to lower the limit of drug detection to fit the therapeutic range even in human serum. Consequently, we prove that the proposed method is suitable for drug monitoring for applications in personalized therapy.

## 2. Experimental Section

### 2.1. Materials

The biosensor was fabricated by using commercially available carbon paste screen-printed electrodes (SPE model DRP-110, purchased from DropSens, Llanera, Spain), made of a graphite working electrode (area, 12.56 mm^2^), a graphite counter electrode and an Ag/AgCl reference electrode. Multi walled carbon nanotubes (MWCNT, diameter 10 nm, length 1–2 μm, with 5% –COOH groups content), purchased in powders from DropSens were diluted in chloroform to the fixed concentration of 1 mg/mL and then sonicated for 30 min in order to obtain a homogeneous suspension breaking macro-aggregates. Cytochromes P4503A4 and 1A2 (from Sigma-Aldrich, St. Gallen, Switzerland), and cytochrome P4502B6 (from BD Bioscience, Franklin Lakes, NJ, USA), were purchased as isozyme microsomes with P450 reductase and cytochrome b5, recombinant, expressed in baculovirus infected insect cells (BTI-TN-5B1-4). Microsomes were given in solution in 100 mM phosphate buffer saline (PBS, from Sigma-Aldrich), at pH 7.4 and used without modifications. All the drugs, cyclophosphamide, ifosfamide, ftorafur and etoposide, were purchased in powder from Sigma-Aldrich. Cyclophosphamide, Ifosfamide, Ftorafur were dissolved in Milli-Q water. Etoposide was dissolved in dimethyl sulfoxide (DMSO) due to its low solubility in water. PBS 100 mM (10×, pH 7.4) and human serum were used as supporting electrolytes. Human serum was purchased from Lonza (Basel, Switzerland) and used without any dilution.

### 2.2. Preparation of Nano-Structured Electrodes

The CNT solution (30 μL) was gradually deposited by drop-casting onto the working electrode. After each single deposition, the chloroform evaporates and the nanotubes lay down on the electrode surface forming a 3D porous nanostructure. The P450 solution (usually 9 μL of solution for each protein layer) was spread onto the CNT-electrode surface and incubated at 4 °C overnight, to allow the protein to be homogeneously adsorbed onto the CNTs-nanostructure. This procedure was repeated for every P450 deposition after having incubated electrodes for 8 h at 4 °C. All the functionalized electrodes were stored at +4 °C and covered with PBS when not used.

### 2.3. Surface Imaging

Morphological analysis of the nano-structured electrodes was carried out using a Philips/FEI XL-30F microscope (Eindhoven, The Netherlands) to acquire Scanning Electron Microscope (SEM) images for bare and nano-structured electrodes. Scanning electron microscope operating in ultra-high resolution mode (UHR), with a working distance in the range 1.5–4.2 mm, was used to analyze the morphology of electrode surface after the modification with MWCNTs and cytochrome P450 solutions. The resolution in UHR mode is 2.5 nm at 1 kV.

### 2.4. Electrochemical Measurements

All electrochemical measurements were performed using a Versastat 3 potentiostat (Princeton Applied Technologies, Oak Ridge, TN, USA). Cyclic voltammograms were acquired under aerobic conditions after having covered the electrode with 100 μL of PBS 100 mM (PBS 10×, pH 7.4) or human serum and adding drug samples at different concentrations. The potential was swept in the range from −600 mV to +300 mV *vs.*Ag/AgCl using a scan rate of 20 mV/s. We use a scan rate of 20 mV/s to control the capacitive current [28]. The procedure described in [29] is used to determine peak current values. Detection limit and sensitivity were the key parameters used for evaluating the measure quality. The sensitivity was determined as the slope of the calibration line which interpolates experimental data. According to existing approaches [30,31], the detection limit was calculated as three-times the signal-noise ratio (*i.e.*, three-times the ratio of the signal standard deviation obtained in the absence of the analyte over the slope of the calibration curve). Both the sensitivity and limit of detection were evaluated inside the linear range of each calibration curve.

## 3. Results and Discussion

### 3.1. Electrode Nanostructuration

**Figure 1.**
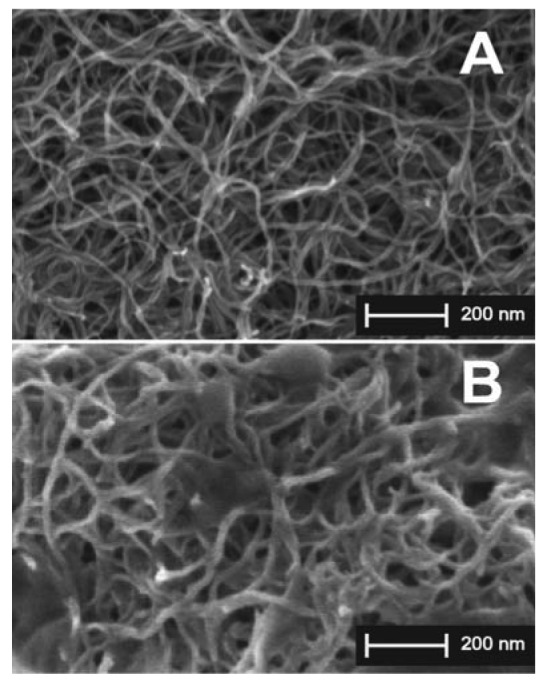
SEM images of graphite electrode after the drop-casting deposition of MWCNTs (**A**), and after the deposition of one layer (**B**) of a solution of cytochrome P4503A4. All images were acquired by using scanning electrode microscope in ultra-high resolution mode. Figure 1(A,B) was acquired with a 100,000× magnification.

Typical scanning electron microscopy (SEM) images of the surface of a graphite electrode after the deposition of 30 μg of MWCNTs (A) and the deposition of one layer (B) of cytochrome P4503A4, are depicted in Figure 1. After the deposition of the CNT-solution (Figure 1(A)), the chloroform evaporates and the nanotubes lay down on the electrode surface forming a 3D porous structure made by agglomerates of wrapped wires randomly spread onto the surface. After the deposition of one layer of protein (Figure 1(B)), an increase in CNT-diameter size (of about 10 ± 4 nm) is observed, demonstrating that nanotubes are completely surrounded by a protein layer. With subsequent depositions of cytochrome P450 solutions, a lot of charging problems occurred (images not shown), causing significant image distortion and compromising the quality of SEM image acquisition, although this phenomenon demonstrates that there is an accumulation of the enzyme on the electrode surface.

The electrochemical response of the sensor was evaluated by cyclic voltammetry measurements under aerobic conditions in 100 μL of PBS 10× (pH 7.4), as the supporting electrolyte. In the absence of the CYP substrate, cytochrome P450 can react with oxygen when a redox potential is applied to the electrode. This reaction can be quantified by analyzing the reduction peaks in cyclic voltammetry measurements. The faradic current associated with the reduction peak is dependent on the quantity of CYP immobilized onto the electrode and the peak potential is influenced by the immobilization technique. The mechanism of the reaction between the immobilized onto the electrode surface CYP and oxygen is outlined in [32]. When the drug (substrate) is added to a buffer solution in presence of oxygen, a further increase in the CYP reduction current is observed.

Current peaks and the related potential positions obtained from cyclic voltammetry acquired in PBS for 1-2-3-4 CYPs depositions are reported in Table 1. The maximum current is shown to increase with the growing number of depositions, demonstrating the improvement of the sensitivity through the multi-layer protein deposition, also confirmed by SEM images. Nanostructuring electrodes with MWCNTs lead to an enhancement in the catalytic current since nanotubes and enzyme molecules are of similar dimensions, promoting the enzyme adsorption without significant loss of its shape or catalytic activity. It is thought that physical adsorption of enzymes onto carbon nanotubes enables a direct electron transfer between the electrode and the active site of the redox-enzyme, minimizing the electron tunneling distance [33]. Moreover, the three-dimensional structure formed by the carbon-nanotubes onto the electrode surface provides a surface area larger than the bare electrode surface, with a consequent increase in the amount of adsorbed enzyme, a deeper penetration of the electrolytes solution and also a larger surface disposable for the redox reaction.

### 3.2. Enzyme Adsorption

In order to investigate the enzyme adsorption onto carbon nanotubes, we performed numerical simulations. With Monte Carlo simulations, we obtained the average diameter of carbon nanotubes after the cytochrome P450 solution has been cast onto carbon nanotubes randomly spread on the graphite surface of screen-printed electrodes.

Crystallographic studies on the binding energy in antigen-antibody complexes [34] revealed that the binding specificity and the physical forces involved in the protein-protein complexes formation can be quantified through the estimation of changes in the Gibbs free energy and in principle, be applied to a wide range of macromolecular systems and protein-surfaces interactions, such as between cytochrome P450 and carbon nanotubes. Similar to studies related to the antigen-antibody interaction, we can write the free energy associated with the protein-CNT complex as a function of different sources of interactions [34]:

(1) $$\Delta G=\Delta H_{\Phi }+\Delta H_{EL}-T\Delta S_{CF}-T\Delta S_{\text{TR}}-T\Delta S_{\text{ID}}.$$

In Equation (1), we have different terms relating to different sources of the antigen-antibody interaction. *ΔH_Φ_* is due to the hydrophobic interactions, *ΔH_EL_* accounts for the Van der Waals forces, *ΔS_CF_* is the variation of the conformational entropy, *ΔS_TR_* is the variation of the entropy associated with the loss of translational and rotational degree of freedom by the protein P450, and *ΔS_ID_* is the variation of the entropy associated with a mole of pure water that stabilizes the cytochrome P450-CNT interaction. The major contributor in Equation (1) is the hydrophobic interactions between the two surfaces from two different molecules (the CYP and the carbon nanotube) that participate in the bond. The term due to the hydrophobic interactions *ΔH_Φ_* may be computed as [34]:

(2) $$\Delta H_{\Phi }=\alpha A_{\text{contact}}.$$

Equation (2) shows that the enthalpy related to hydrophobic interactions is proportional to the contact area between the antibody and the antigen. This enthalpy is related to the solvent-accessible surface of the side chains of the two structures forming the complex. Direct experiments have shown that 1 Å^2^ of buried surface corresponds to 104.5 J/mol in case of antibody/antigen hydrophobic interactions. Consequently, the coefficient of proportionality in Equation (2) has been empirically determined as:

(3) $$\alpha =-104.5\;kJ/mol\;nm^{2}.$$

**Figure 2.**
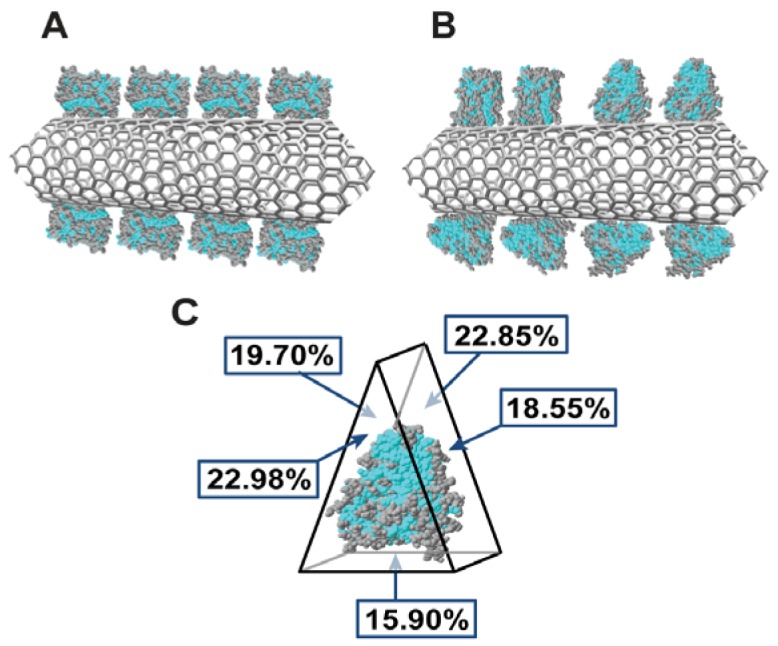
Orientation of microsomal cytochrome P450 3A4 on carbon nanotubes and its crystallographic structure. Figure 2(A) represents the most likely orientation of cytochrome onto the carbon nanotube according to the percentage of hydrophobic sites reported in Figure 2(C). A distinct triangular prismatic shape is evident, while the hydrophobic residues are highlighted in blue. For the construction of the model for Monte Carlo simulations, it has been assumed that cytochrome molecules mainly expose their larger face (*i.e.*, the lateral face, Figure 2(A)), for hydrophobic interaction with carbon nanotubes walls, since it is not possible to predict the right protein orientation onto the nanotube walls. Figure 2(B) represents four other possible orientations of cytochrome onto the carbon nanotube.

In order to roughly estimate the bond strength between the P450 and the carbon nanotube we can compare the enthalpy estimated by (2) with those for the antigen-antibody interactions. Typical contact area in the antigen-antibody interaction ranges from 150 Å^2^ up to 690 Å^2^. Consequently, Equations (2) and (3) describe a range for the hydrophobic enthalpy from −16 kJ/mol up to −74 kJ/mol. It is known from crystallographic studies that cytochrome P450s have a triangular prismatic shape (Figure 2) with approximate dimension of 4.5–5 nm × 5–6.5 nm and a thickness of 3.5–4.5 nm [35,36]. Thus, for cytochrome P450-CNT interaction we can estimate a contact area in the range 272 Å^2^–378 Å^2^, which corresponds to a hydrophobic enthalpy between −28 kJ/mol and −40 kJ/mol. These enthalpies are in the same order of magnitude of antigen-antibody interaction, demonstrating that hydrophobic interactions play an important role in determining the cytochrome P450 attachment onto the CNT surface.

The interaction between cytochrome P450 and carbon nanotubes is difficult to predict, since cytochrome presents on the surface several hydrophobic sites formed by *N*-terminal domain residues, hydrophobic residues [37], and also by the *C*-terminal end of the F-G loop [38], whereas other surfaces of the protein are mostly hydrophilic. It is known [39] that cytochrome basically interacts with its reaction partners as well as with biological membranes, through electrostatic forces and hydrophobic interactions. In both cases, the establishment of these interactions induces functionally relevant changes in the cytochrome structure [37].

Several works [37,38,40] have reported that cytochrome P450 appears to be mono-facially attached to the membrane via a broad hydrophobic surface adjacent to the anchor provided by a transmembrane helix at the *N*-terminus, thus becoming partially buried in the membrane phospholipidic bilayer [38]. Studies with cytochrome P450 immobilized through physical adsorption onto gold surfaces [35], or onto a Self-Assembled-Monolayer (SAM) of methyl-terminated alkanethiols [39], demonstrated that hydrophobic interactions are generated between hydrophobic residues of the protein and the hydrophobic surfaces. These hydrophobic interactions lead to a non-specific and largely random orientation of the immobilized enzymes, even if it has been demonstrated that cytochromes preferentially adopt a largely uniform orientation that is favorable for a rapid electron transfer [39].

Other studies [41] reported that enzymes, such as cytochrome c are suitable for self-assembly onto metal surfaces due to their specific and non-uniform charge surface distribution and, in particular, it has been demonstrated that proteins, such as streptavidin have the tendency to self-organize around multi-walled carbon-nanotubes due to the amine groups of protein residues, which interacts with the hydrophobic sites of CNTs [42].

For Monte Carlo simulations, we assumed that a high percentage of CNTs expose their walls for protein adsorption. The agglomerates of CNT after they have been drop casted onto the electrode surface are shown in Figure 1(A). This behavior, described in [43], is due to the fact that the ends of carbon nanotubes are quite hydrophilic, but the walls, which comprise the majority of the tube, are highly hydrophobic. Adsorbed CNTs are not completely embedded into the electrode material as in carbon-nanotubes paste electrodes [44,45].

For protein adsorption, we reasonably assumed that the enzyme generates a self-assembled monolayer with a largely random orientation. In this case, the most important contribution to the Gibbs free energy changes (associated with the non-covalent cytochrome-CNT complex formation), is the hydrophobic effect, which is directly proportional to the solvent-accessible surface area at the solvent-solute interface. The difference between solvent-accessible surfaces of free and complex molecules provides the contact area of the complex. Therefore, for Monte Carlo simulations we assume that the probability of cytochrome attachment onto a specific hydrophobic site of CNT wall is directly proportional to its contact area (Figure 2(C)) and also that the adsorption of molecules of cytochrome P450 3A4 onto CNTs, considered as substrate, mimics the formation of the self-assembled monolayers (SAM).

Table 2 reports results from Monte Carlo simulations compared with the measurements obtained from SEM images, demonstrating the capability of this model to describe the physical adsorption of cytochrome onto multi-walled carbon-nanotubes.

Moreover, with these simulations we obtained an average thickness for the enzyme adsorbed on the nanotube between 3 nm and 6 nm. These values are consistent with the size of a cytochrome P450 molecule, also considering the possible different orientations when adsorbed. This thickness range demonstrates that a single protein layer homogeneously covers the carbon nanotube walls (as in the SEM image reported in Figure 1(B)).

### 3.3. Bio-Detection Mechanism

**Figure 3.**
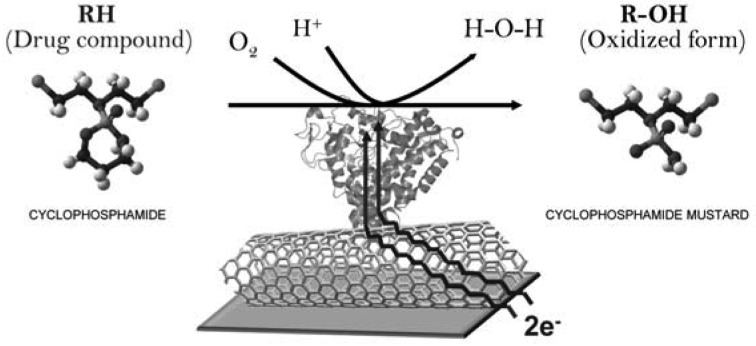
Schematic representation of the drug detection mechanism enhanced by carbon nanotubes. The example illustrates the reaction of hydroxylation for the anti-cancer drug cyclophosphamide.

The biosensing mechanism is mainly based on the biochemical reaction plotted in Figure 3 where a cytochrome P450 transforms the redox form of a drug (cyclophosphamide in the example) in its oxidized form by using one oxygen molecule and two electrons. In voltammetric measurements, the two electrons, which in nature are provided to the enzyme by NADPH molecules [3], are supplied by the flowing current coming from the electrode. The detection mechanism is the measurement of the current produced at the electrode surface due to the redox reaction of the enzyme when a substrate is present in the sample. The cyclic voltammograms show cathodic and anodic current peaks that correspond to the electrochemical signal registered during the redox reaction catalyzed by the cytochrome.

**Figure 4.**
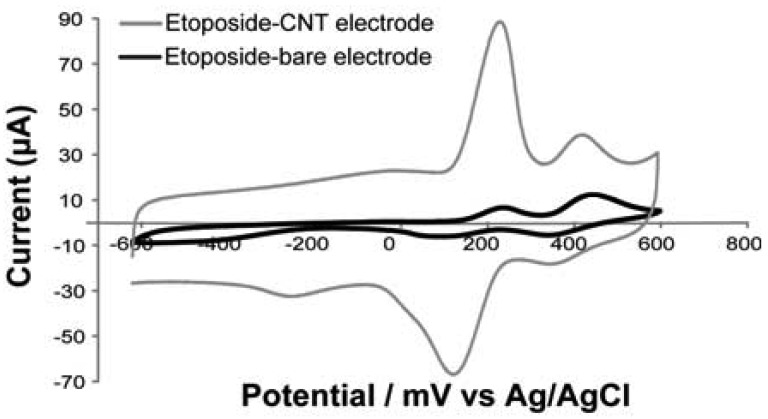
Comparison between cyclic voltammograms obtained with bare electrode (black curve) and CNT-nanostructured electrode (grey curve), in presence of etoposide 100 μM. The peak at around −200 mV is due to the oxygen moieties derived from carbon-nanotubes as reported in [5,47]. Two oxidation peaks at +220 mV and +450 mV and two reduction peaks at +150 mV and +350 mV are visible. These data confirm the peaks reported in literature [48], obtained through etoposide cyclic voltammetry at glassy carbon electrode.

Multi-walled carbon nanotubes are used for enhancing the electron transfer [17]. Electronic properties of MWCNTs suggest that they can promote electron-transfer reactions when used as the electrode material in electrochemical reactions. Other studies demonstrated that CNTs can also enhance the electrochemical reactivity of proteins or enzymes with retention of their biocatalytic activity [17,20], thus making them suitable to be integrated into biological sensors [46]. The CNTs current-enhancement effect is illustrated in Figure 4, where we compare the voltammetric response recorded for a CNT-nanostructured electrode and for a bare electrode, in presence of Etoposide 100 μM. As can be seen, two well-defined oxidation peaks and two reduction peaks are observable in the voltammetric response for the CNT electrode, while the bare electrode exhibits a voltammogram with a smaller capacitive current and reduced current peaks. In Figure 4 the improvement of analytic performances with the nanostructures in comparison with the bare electrode is clearly shown. Furthermore, multi-walled carbon nanotubes exhibit good electronic communication with redox proteins immobilized on the electrode surface [17,20].

### 3.4. Drug Detection in Buffer Solution and Human Serum

**Figure 5.**
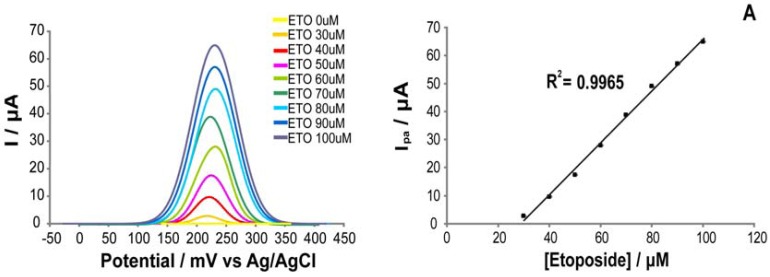

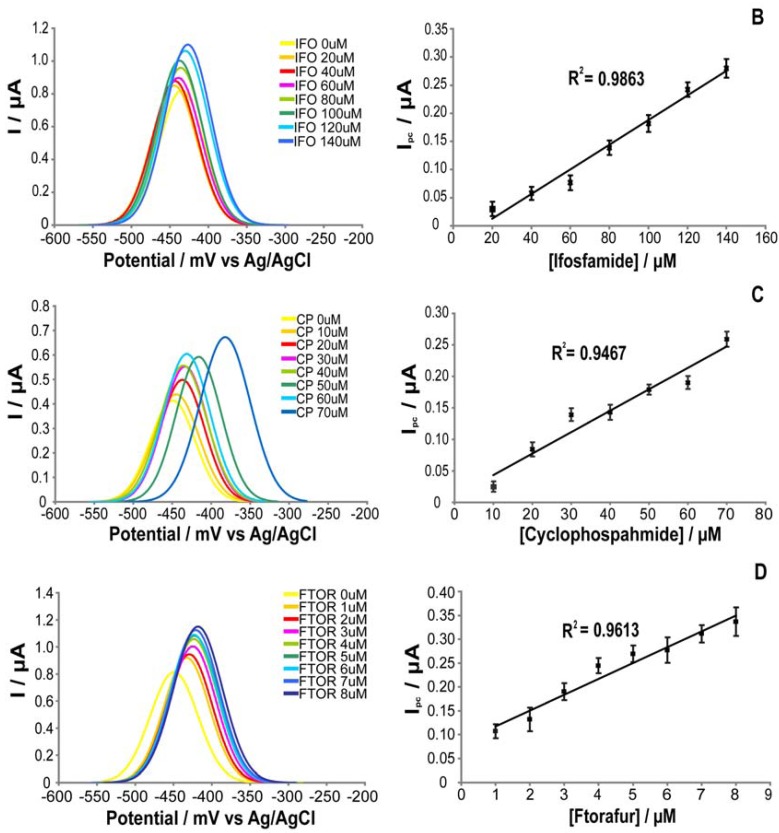
Cyclic voltammetric responses (on the left) and calibration curves (on the right), obtained from cyclic voltammetric measurements in PBS with increasing aliquots of drugs: CNT-electrode for etoposide (**A**), cytochrome P4503A4 for ifosfamide (**B**), cytochrome P4502B6 for cyclophosphamide (**C**), and P4501A2 for ftorafur (**D**). For the CNT-electrode the zoom in the oxidation peak at 220 mV is shown (**A**, on the left). For the electrodes modified with cytochromes we report the zoom of the reduction peak at −450 mV (**B, C**, and **D**, on the left). The measured drugs concentration falls in their pharmacological range. The voltammograms were acquired with drugs dissolved in PBS and at the scan rate of 20 mV/s.

After having considered several drugs and the related pharmacological range, we selected four compounds: cyclophosphamide, etoposide, ftorafur, and ifosfamide. These four drugs have been chosen because they have sufficiently high and wide pharmacological ranges, compatible with the detection limit of the measurement setup. To investigate the feasibility of our system for drug detection, we tested it with these drugs using three different P450 isoforms (CYP1A2, CYP2B6 and CYP3A4). The measurements were performed in PBS (pH 7.4) and in human serum. Voltammetric responses for each cytochrome-drug pairs were firstly acquired in absence of drug and then in presence of the drug in increasing concentration within their pharmacological range. The voltammetric responses to increasing aliquots of drugs and the calibration curves of the sensors are reported in Figure 5. The sensor potential for measuring the presence of drugs with different P450 isoforms is demonstrated in Table 3, which compares the results obtained in PBS and in human serum. Detection limit and sensitivity were the key parameters used to evaluate the quality of measurements. Detection limit and sensitivity have been evaluated within the linear range of the calibration curves. The voltammograms exhibited two cathodic peak regions at potentials centered around −330 mV and −450 mV, respectively. The current responses reported in Figure 5 have been measured at a cathodic peak potential of around −450 mV for each CYP-electrode (the corresponding data of sensitivities and limits of detection are not reported). The current responses reported in Table 3 have been measured at a cathodic peak potential around −330 mV *vs.*Ag/AgCl for each drug in the pharmacological range. For the CNT-electrode (for Etoposide detection), the current response reported in Figure 5(A) has been measured at an anodic peak potential of around +220 mV. Moreover, we obtained values of detection limits within the therapeutic range for each drug, and also sensitivities that are comparable with the results shown by other authors with CYP-based sensors [6–8,49–54].

As expected, the system exhibits better performance in PBS than in serum. However, for both cases the limits of detection are within the pharmacological ranges for all the tested drugs, proving the feasibility of P450-based biosensor for measurements in human plasma. In these results it is evident that current peaks appear to be partially or totally modified by the presence of serum proteins, as another work has also showed [59]. The decrease in sensitivity obtained in serum can be reasonably attributed to the plasma proteins, such as albumin, bilirubin or hemoglobin, which can bind and interact with drug molecules, reducing the free drug concentration at the electrode surface, as already reported [60,61]. In case of ftorafur for example, it was reported that it weakly binds only to plasma protein (about 22%) [62], while etoposide is one of the few anti-cancer drugs that strongly binds (>90%) to plasma proteins [63]. Cyclophosphamide and ifosfamide exhibit relatively low protein binding. The active metabolites of these drugs, however, have been found to significantly bind blood protein, such as albumin and hemoglobin, up to 40% [60]. Plasma proteins can also be not-specifically adsorbed on the electrode surface and partially block the binding site of the enzyme, thus further reducing the signal.

### 3.5. Multiple Drug Detection

**Figure 6.**
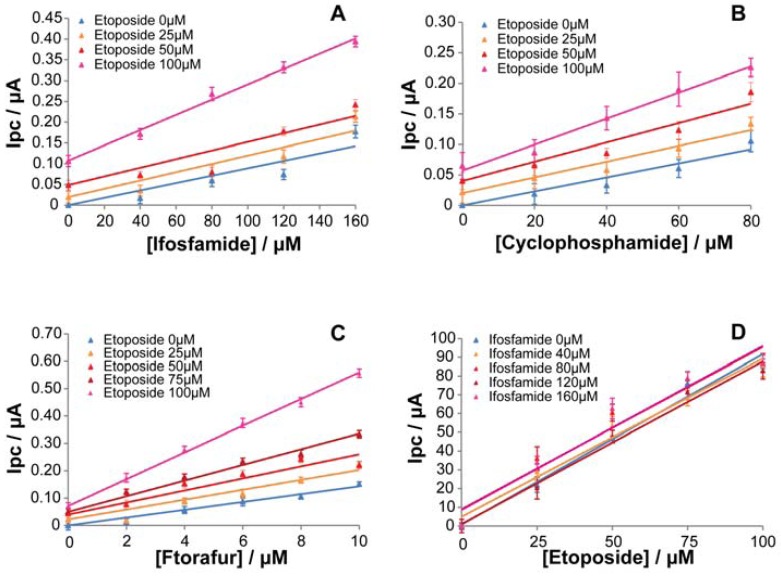
Calibration curve families. Cyclic voltammetric measurements, in PBS, obtained for all drugs in their pharmacological range and etoposide at fixed concentrations (0–25–50–75–100 μM). Cytochrome P4503A4 was used for ifosfamide (**A**), cytochrome P4503A4 for cyclophosphamide (**B**), and cytochrome P4501A2 for ftorafur (**C**). Calibration curves for etoposide detection in its pharmacological range obtained with a CNT-electrode in presence of ifosfamide at fixed concentrations (0–40–80–120–160 μM) are illustrated in (**D**).

The possibility to detect the presence of two drugs in the sample has been investigated with cyclic voltammetric measurements obtained by the addition of cyclophosphamide, ifosfamide, and ftorafur in the presence of etoposide at fixed concentrations (0–25–50–75–100 μM). According to the possible chemotherapy regimens administered for the treatment of breast-cancer, three sets of paired drugs were tested (cyclophosphamide-etoposide, ifosfamide-etoposide, and ftorafur-etoposide), since they were the only possible drug pairs that may interact because they are metabolized by the same isoform. The results are reported in Figure 6, which compares the families of calibration curves obtained for three different P450-CNT electrodes: CYP3A4 for ifosfamide (A), CYP3A4 for cyclophosphamide (B), and CYP1A2 for ftorafur (C). A well-defined increase in the sensitivity with etoposide concentration is visible in each case. This behavior is probably due to a partial hetero-activation effect of etoposide on CYP3A4 and CYP1A2-mediated metabolism of the other drugs [64,65]. Several CYP isoforms, including 3A4, 1A2, 2E1, 2D6, and 2C9, have been reported to exhibit allotropic kinetics *in vitro* [9]. This atypical kinetic behavior is exhibited by enzymes with multiple substrate recognition sites, such as CYPs, and it is basically due to conformational or chemical changes that occur in the active site of the enzyme after binding a first substrate. These changes in the conformation of enzyme tertiary and quaternary structures result in the enzyme catalytic activity alteration which can affect the metabolism of a second substrate [10]. In Figure 6(D), each calibration curve has been obtained by cyclic voltammetry measurements at increasing etoposide concentration with a fixed concentration of ifosfamide. This plot shows that the increase in ifosfamide concentration does not affect Etoposide detection and this is indicated by the overlap of the calibration curves. Figure 6(D) confirms the specificity of the CNT electrode in detecting etoposide, even in presence of a second drug (similar curves have been obtained with CP and ftorafur, data not shown). Consequently, measuring etoposide concentration with a CNT-electrode would give us the possibility to indirectly obtain the concentration of the second compound (cyclophosphamide, ifosfamide or ftorafur), that might be present in the same blood sample.

## 4. Conclusions

In this work we demonstrated that a biosensor based on cytochrome P450 (CYP1A2, CYP2B6 and CYP3A4) and carbon-nanotubes enable the identification of different anti-cancer drugs, thus providing an innovative solution for point-of-care drug monitoring. The results show sensitivities in the range of 8–925 nA/μM. With these high sensitivities, we could perform a selective electrochemical detection of drugs in their therapeutic window. We showed how proteins arrange themselves in a monolayer onto the carbon-nanotubes surface, and we verified this behavior both with Monte Carlo simulations and SEM analysis. The use of three different CYP isoforms can provide specific drug detection, overcoming the lack of specificity for a single substrate typical of cytochrome P450. In this work, we perform a preliminary study for the realization of an array-sensor based on cytochrome P450. The main goal was to demonstrate the suitability of such sensor for detecting anti-cancer drugs within the pharmacological range in human serum. We also demonstrate that detection of two drugs at the same time can be achieved with an accurate selection of the isoform as enzyme probe according to the drug to be detected. Future work includes the realization of an array-based sensor, a study of kinetics and drug-drug interaction on the same enzyme, and a system-level analysis of the sensor in order to consider possible interferences. We conclude that these P450-based sensors represent a powerful nano-technology able to perform drug detection directly in human plasma, which can be successfully used to develop point-of care and low cost devices for personalized therapy.

## Figures and Tables

**Table 1.** Current peaks and the related potential positions obtained with cyclic voltammetry in PBS for different number of CYP3A4 depositions.

***N° Layers***	***Potential (mV)***	***Max current value (nA)***
1	−414 ± 12	18 ± 97
2	−390.7 ± 4.7	727 ± 13
3	−384.0 ± 4.6	1168 ± 20
4	−391.4 ± 4.4	1519 ± 23

**Table 2.** Average diameters of MWCNTs covered by a cytochrome P4503A4 monolayer, obtained by measurements on SEM images and Monte Carlo simulations.

***Measurements SEM images***	20 ± 3 nm
***Monte Carlo simulations***	20 ± 4 nm

**Table 3.** Performances of electrodes nanostructured with CNTs and different CYP isoforms for drug detection.

***Drugs***	***Pharmacological range (μM)***	***P450 enzyme***	Sensitivity (nA/μM*mm^2^)		***Detection limit (μM)***	

			**PBS**	**Serum**	**PBS**	**Serum**
Cyclophosphamide	2.68–76.6 [55]	2B6	1.0 ± 0.0	0.3 ± 0.1	2.4 ± 0.0	13.8 ± 4.3
		3A4	0.6 ± 0.0	0.3 ± 0.0	4.9 ± 0.3	10.8 ± 1.8
Ifosfamide	10–160 [56]	2B6	1.2 ± 0.0	0.1 ± 0.0	2.8 ± 0.1	40 ± 18
		3A4	1.6 ± 0.0	0.4 ± 0.0	2.0 ± 0.0	7.1 ± 0.5
Ftorafur	1–10 [57]	1A2	8.8 ± 0.7	3.9 ± 0.5	0.7 ± 0.1	0.8 ± 0.1
Etoposide	33.98–101.94 [58]	-	73.7 ± 0.0	9.1 ± 0.1	0.05 ± 0.0	0.5 ± 0.0

## References

[1] Bistolas N., Wollenberger U., Jung C., Scheller F.W. (2005). Cytochrome p450 biosensors-a review. Biosens. Bioelectron..

[2] Johnson D.L., Lewis B.C., Elliot D.J., Miners J.O., Martin L.L. (2005). Electrochemical characterisation of the human cytochrome p450 cyp2c9. Biochem. Pharmacol..

[3] Guengerich F.P. (2008). Cytochrome p450 and chemical toxicology. Chem. Res. Toxicol..

[4] Shumyantseva V.V., Bulko T.V., Usanov S.A., Schmid R.D., Nicolini C., Archakov A.I. (2001). Construction and characterization of bioelectrocatalytic sensors based on cytochromes p450. J. Inorg. Biochem..

[5] Carrara S., Shumyantseva V., Archakov A., Samorì B. (2008). Screen-printed electrodes based on carbon nanotubes and cytochrome p450scc for highly sensitive cholesterol biosensors. Biosens. Bioelectron..

[6] Joseph S., Rusling J.F., Lvov Y.M., Friedberg T., Fuhr U. (2003). An amperometric biosensor with human cyp3a4 as a novel drug screening tool. Biochem. Pharmacol..

[7] Liu S., Peng L., Yang X., Wu Y., He L. (2008). Electrochemistry of cytochrome p450 enzyme on nanoparticle-containing membrane-coated electrode and its applications for drug sensing. Anal. Biochem..

[8] Iwuoha E.I., Wilson A., Howel M., Mathebe N.G.R., Montane Jaime K., Narinesingh D., Guiseppi Elie A. (2005). Cytochrome p4502d6 (cyp2d6) bioelectrode for fluoxetine. Anal. Lett..

[9] Atkins W.M. (2005). Non-michaelis-menten kinetics in cytochrome p450-catalyzed reactions. Annu. Rev. Pharmacol. Toxicol..

[10] Houston J.B., Galetin A. (2005). Modelling atypical cyp3a4 kinetics: Principles and pragmatism. Arch. Biochem. Biophys..

[11] He P., Hu N., Rusling J.F. (2003). Driving forces for layer-by-layer self-assembly of films of sio2 nanoparticles and heme proteins. Langmuir.

[12] Shumyantseva V.V., Carrara S., Bavastrello V., Jason Riley D., Bulko T.V., Skryabin K.G., Archakov A.I., Nicolini C. (2005). Direct electron transfer between cytochrome p450scc and gold nanoparticles on screen-printed rhodium-graphite electrodes. Biosens. Bioelectron..

[13] Wu Y., Hu S. (2007). Biosensors based on direct electron transfer in redox proteins. Microchim. Acta.

[14] Luo X., Morrin A., Killard A.J., Smyth M.R. (2006). Application of nanoparticles in electrochemical sensors and biosensors. Electroanalysis.

[15] Wang J. (2005). Nanomaterial-based electrochemical biosensors. Analyst.

[16] Zhang X., Guo Q., Cui D. (2009). Recent advances in nanotechnology applied to biosensors. Sensors.

[17] Wang J. (2005). Carbon-nanotube based electrochemical biosensors: A review. Electroanalysis.

[18] Zhao Q., Gan Z., Zhuang Q. (2002). Electrochemical sensors based on carbon nanotubes. Electroanalysis.

[19] Lin Y., Lu F., Tu Y., Ren Z. (2003). Glucose biosensors based on carbon nanotube nanoelectrode ensembles. Nano Lett..

[20] Gooding J.J. (2005). Nanostructuring electrodes with carbon nanotubes: A review on electrochemistry and applications for sensing. Electrochim. Acta.

[21] Hortobagyi G.N. (1998). Treatment of breast cancer. N. Engl. J. Med..

[22] Smith I.C., Heys S.D., Hutcheon A.W., Miller I.D., Payne S., Gilbert F.J., Ah-See A.K., Eremin O., Walker L.G., Sarkar T.K. (2002). Neoadjuvant chemotherapy in breast cancer: Significantly enhanced response with docetaxel. J. Clin. Oncol..

[23] Roy P., Yu L.J., Crespi C.L., Waxman D.J. (1999). Development of a substrate-activity based approach to identify the major human liver p-450 catalysts of cyclophosphamide and ifosfamide activation based on cdna-expressed activities and liver microsomal p-450 profiles. Drug Metab. Dispos..

[24] Huang Z., Roy P., Waxman D.J. (2000). Role of human liver microsomal CYP3A4 and CYP2B6 in catalyzing *N*-dechloroethylation of cyclophosphamide and ifosfamide. Biochem. Pharmacol..

[25] Komatsu T., Yamazaki H., Shimada N., Nakajima M., Yokoi T. (2000). Roles of cytochromes p450 1a2, 2a6, and 2c8 in 5-fluorouracil formation from tegafur, an anticancer prodrug, in human liver microsomes. Drug Metab. Dispos..

[26] Komatsu T., Yamazaki H., Shimada N., Nagayama S., Kawaguchi Y., Nakajima M., Yokoi T. (2001). Involvement of microsomal cytochrome p450 and cytosolic thymidine phosphorylase in 5-fluorouracil formation from tegafur in human liver. Clin. Cancer. Res..

[27] Zhuo X., Zheng N., Felix C.A., Blair I.A. (2004). Kinetics and regulation of cytochrome p450-mediated etoposide metabolism. Drug Metab. Dispos..

[28] Chen J.H., Li W.Z., Wang D.Z., Yang S.X., Wen J.G., Ren Z.F. (2002). Electrochemical characterization of carbon nanotubes as electrode in electrochemical double-layer capacitors. Carbon.

[29] Carrara S., Cavallini A., Garg A., de Micheli G. Dynamical Spot Queries to Improve Specificity in p450s Based Multi-Drugs Monitoring.

[30] Lavagnini I., Antiochia R., Magno F. (2007). A calibration-base method for the evaluation of the detection limit of an electrochemical biosensor. Electroanalysis.

[31] Mocak J. (1997). A statistical overview of standard (iupac and acs) and new procedures for determining the limits of detection and quantification: Application to voltammetric and stripping techniques. Pure Appl. Chem..

[32] Shumyantseva V.V., Ivanov Y.D., Bistolas N., Scheller F.W., Archakov A.I., Wollenberger U. (2004). Direct electron transfer of cytochrome p450 2b4 at electrodes modified with nonionic detergent and colloidal clay nanoparticles. Anal. Chem..

[33] Lyons M.E.G., Keeley G.P. (2008). Carbon nanotube based modified electrode biosensors. Part 1. Electrochemical studies of the flavin group redox kinetics at swcnt/glucose oxidase composite modified electrodes. Int. J. Electrochem. Sci..

[34] Novotny J., Bruccoleri R.E., Saul F.A. (1989). On the attribution of binding energy in antigen-antibody complexes mcpc 603, d1.3, and hyhel-5. Biochemistry.

[35] Davis J.J., Djuricic D., Lo K.K., Wallace E.N., Wong L.L., Hill H.A. (2000). A scanning tunnelling study of immobilised cytochrome p450cam. Faraday Discuss.

[36] Ravichandran K.G., Boddupalli S.S., Hasermann C.A., Peterson J.A., Deisenhofer J. (1993). Crystal structure of hemoprotein domain of p450bm-3, a prototype for microsomal p450's. Science.

[37] Rivas L., Murgida D.H., Hildebrandt P. (2002). Conformational and redox equilibria and dynamics of cytochrome c immobilized on electrodes via hydrophobic interactions. J. Phys. Chem. B.

[38] Williams P.A., Cosme J., Sridhar V., Johnson E.F., McRee D.E. (2000). Microsomal cytochrome p450 2c5: Comparison to microbial p450s and unique features. J. Inorg. Biochem..

[39] Murgida D.H., Hildebrandt P. (2004). Electron-transfer processes of cytochrome c at interfaces. New insights by surface-enhanced resonance raman spectroscopy. Acc. Chem. Res..

[40] de Montellano P.R.O. (2005). Cytochrome p450.

[41] Macdonald I.D.G., Smith W.E. (1996). Orientation of cytochrome c adsorbed on a citrate-reduced silver colloid surface. Langmuir.

[42] Bradley K., Briman M., Star A., Grüner G. (2004). Charge transfer from adsorbed proteins. Nano Lett..

[43] Boero C., Carrara S., Del Vecchio G., Calza L., de Micheli G. (2011). Highly sensitive carbon nanotube-based sensing for lactate and glucose monitoring in cell culture. IEEE Trans. Nanobiosci..

[44] Luque G.L., Ferreyra N.F., Rivas G.A. (2006). Glucose biosensor based on the use of a carbon nanotube paste electrode modified with metallic particles. Microchim. Acta.

[45] Rubianes M.D., Rivas G.A. (2003). Carbon nanotubes paste electrode. Electrochem. Commun..

[46] Wang J., Li M., Shi Z., Li N., Gu Z. (2002). Direct electrochemistry of cytochrome c at a glassy carbon electrode modified with single-wall carbon nanotubes. Anal. Chem..

[47] Banks C.E., Davies T.J., Wildgoose G.G., Compton R.G. (2005). Electrocatalysis at graphite and carbon nanotube modified electrodes: Edge-plane sites and tube ends are the reactive sites. Chem. Commun..

[48] Holthuis J.J.M., van Oort W.J., Römkens F.M.G.M., Renema J., Zuman P. (1985). Electrochemistry of podophyllotoxin derivatives: Part I. Oxidation mechanism of etoposide (VP 16-213). J. Electroanal. Chem. Interfacial Electrochem..

[49] Antonini M., Ghisellini P., Pastorino L., Paternolli C., Nicolini C. (2003). Preliminary electrochemical characterisation of cytochrome p4501a2-clozapine interaction. IEE Proc. Nanobiotechnol..

[50] Shumyantseva V.V., Ivanov Y.D., Bistolas N., Scheller F.W., Archakov A.I., Wollenberger U. (2004). Direct electron transfer of cytochrome p450 2b4 at electrodes modified with nonionic detergent and colloidal clay nanoparticles. Anal. Chem..

[51] Peng L., Yang X., Zhang Q., Liu S. (2008). Electrochemistry of cytochrome p450 2b6 on electrodes modified with zirconium dioxide nanoparticles and platin components. Electroanalysis.

[52] Iwuoha E., Ngece R., Klink M., Baker P. (2007). Amperometric responses of cyp2d6 drug metabolism nanobiosensor for sertraline: A selective serotonin reuptake inhibitor. IET Nanobiotechnol..

[53] Fantuzzi A., Fairhead M., Gilardi G. (2004). Direct electrochemistry of immobilized human cytochrome p450 2e1. J. Am. Chem. Soc..

[54] Ignaszak A., Hendricks N., Waryo T., Songa E., Jahed N., Ngece R., Al-Ahmed A., Kgarebe B., Baker P., Iwuoha E.I. (2009). Novel therapeutic biosensor for indinavir—A protease inhibitor antiretroviral drug. J. Pharm. Biomed. Anal..

[55] Juma F.D. (1979). Pharmacokinetics of cyclophosphamide and alkylating activity in man after intravenous and oral administration. Br. J. Clin. Pharmacol..

[56] Singer J.M. (1998). The pharmacokinetics and metabolism of ifosfamide during bolus and infusional administration: A randomized cross-over study. Br. J. Cancer.

[57] Liu K. (2010). Determination of tegafur, 5-fluorouracil, gimeracil and oxonic acid in human plasma using liquid chromatography-tandem mass spectrometry. J. Pharm. Biomed. Anal..

[58] Hande K.R. (1984). Pharmacokinetics of high-dose etoposide (VP-16-213) administered to cancer patients. Cancer Res..

[59] Carrara S., Cavallini A., Erokhin V., de Micheli G. (2011). Multi-panel drugs detection in human serum for personalized therapy. Biosens. Bioelectron..

[60] Noort D., Hulst A., Jansen R. (2002). Covalent binding of nitrogen mustards to the cysteine-34 residue in human serum albumin. Arch. Toxicol..

[61] Sulkowska A. (2002). Interaction of drugs with bovine and human serum albumin. J. Mol. Struct..

[62] Yoko S., Yoshiji T., Yasufumi S., Manabu H., Teruyoshi M., Yukihiko U. (1985). Prediction of ftorafur disposition in rats and man by a physiologically based pharmacokinetic model. Int. J. Pharm..

[63] Stewart C.F., Fleming R.A., Arbuck S.G., Evans W.E. (1990). Prospective evaluation of a model for predicting etoposide plasma protein binding in cancer patients. Cancer Res..

[64] Kawashiro T., Yamashita K., Zhao X.-J., Koyama E., Tani M., Chiba K., Ishizaki T. (1998). A study on the metabolism of etoposide and possible interactions with antitumor or supporting agents by human liver microsomes. J. Pharmacol. Exp. Ther..

[65] Chang T.K.H., Yu L., Maurel P., Waxman D.J. (1997). Enhanced cyclophosphamide and ifosfamide activation in primary human hepatocyte cultures: Response to cytochrome p-450 inducers and autoinduction by oxazaphosphorines. Cancer Res..

